# Extracellular vesicles in ovarian cancer chemoresistance, metastasis, and immune evasion

**DOI:** 10.1038/s41419-022-04510-8

**Published:** 2022-01-18

**Authors:** Wanjia Tian, Ningjing Lei, Junying Zhou, Mengyu Chen, Ruixia Guo, Bo Qin, Yong Li, Lei Chang

**Affiliations:** 1 Department of Obstetrics and Gynecology, The First Affiliated Hospital of Zhengzhou University, Zhengzhou, Henan China; 2 School of Basic Medical Sciences, Zhengzhou University, Zhengzhou, Henan China; 3 Translational Medical Center, The First Affiliated Hospital of Zhengzhou University, Zhengzhou, Henan China; 4 Cancer Care Centre, St George Hospital, Kogarah, NSW Australia; 5 St George and Sutherland Clinical School, Faculty of Medicine and Health, UNSW Sydney, Sydney, NSW Australia

**Keywords:** Cancer microenvironment, Translational research

## Abstract

Chemoresistance and metastasis are the major challenges for the current ovarian cancer treatment. Understanding the mechanisms of ovarian cancer progression and metastasis is critically important for developing novel therapies. The advances in extracellular vesicles (EVs) research in recent years have attracted extensive attention. EVs contain a variety of proteins, RNAs, DNAs, and metabolites. Accumulating evidence indicates that ovarian cancer cells secrete a large amount of EVs, playing an important role in tumor progression and recurrence. In the microenvironment of ovarian tumor, EVs participate in the information transmission between stromal cells and immune cells, promoting the immune escape of ovarian cancer cells and facilitating cancer metastasis. Here, we review the recent advances of EVs in chemoresistance, mechanisms of metastasis, and immune evasion of ovarian cancer. Furthermore, we also discuss the challenges of EV research and future application of EVs as promising biomarker sources in response to therapy and in therapy-delivery approaches for ovarian cancer patients.

## Facts

- Chemoresistance, metastasis, and immune evasion are the main challenges in ovarian cancer (OC) clinical treatment.
- EV cargo can regulate the progress of chemoresistance in OC and monitor chemoresistance.
- EVs are thought to stimulate angiogenesis, extracellular matrix remodeling, establish premetastatic niches, inhibit immune response, and promote tumor metastasis.
- The OC-derived EVs inhibit the activation of dendritic cells (DCs), induce the polarization of macrophages, inhibit the cytotoxicity of natural killer (NK) cells, and regulate the function of T cells.
- The OC-derived EVs have the potential application for OC immunotherapy or combination therapeutic development.
- EVs are a source for candidate biomarkers for predicting and monitoring therapeutic drug response of OC patients.
- The EV is proposed as a potential therapeutic target or carrier to reverse OC chemoresistance.

## Open questions

- How EVs mediate chemoresistance in OC?
- Which mechanisms are mediated by EVs to promote OC metastasis?
- What is the role of EVs in the regulation of tumor microenvironment in OC?
- What role do EVs play in OC immune evasion?
- How can EVs be used in OC immunotherapy?
- How to use EVs to improve the dilemma of OC clinical treatment?
- How can EVs be utilized as a biomarker to predict and monitor the chemoresistance in OC patients?
- How can EVs be applied as a drug carrier or a therapeutic target in the treatment of chemoresistant OC?
- What is the future direction for EVs-based OC therapy?

## Introduction

Ovarian cancer (OC) is one of the three common cancers of the female reproductive system. The number of new worldwide cases of OC reached 313,959 in 2020 with 207,252 deaths recorded that year [1]. US statistics from 2020 show that there were 21,750 new OC cases, accounting for about 2.4% of all new cancers in women, and 5% of the 13,940 of female cancer deaths [2]. With the large population base in China, approximately 55,342 women were diagnosed with OC in 2020, accounting for approximately 17.6% of global OC cases, and approximately 37,519 patient deaths, accounting for approximately 18.1% of global OC cases [3]. Since OC develops deeply in the pelvic cavity, and clinical manifestations are limited in the early stage, most OC patients are diagnosed after significant disease progression has occurred. Moreover, owing to its high recurrence rate after resection and common chemoresistance [4], the mortality rate of OC is the highest of all malignant gynecological tumors [5].

OC is classified into more than 15 different molecular and pathological subtypes, which complicates rational treatment decisions [6]. At present, clinical management of OC is mainly based on maximum cytoreductive surgery and combinatorial chemotherapy with cisplatin and paclitaxel [5]. Recently, the use of anti-angiogenic agents and poly-ADP-ribose polymerase (PARP) inhibitors for maintenance therapy draws a lot of attention [7]. Olaparib, a PARP inhibitor related to DNA repair, has shown significant clinical benefit to OC patients with BRCA mutation [8]. Clinical studies have shown that the median progression-free survival (PFS) of patients with OC in the Olaparib group reached 19.1 months, which was higher than 5.5 months in the placebo group, and the median overall survival (OS) increased from 38.8 months to 51.7 months [9, 10]. Nonetheless, most treated patients will eventually suffer from tumor relapse and metastasis. Moreover, relapsed patients develop drug resistance and suffer serious treatment side effects, and eventually die, with the most common cause of death being intestinal obstruction [5, 11–13]. Different targeted therapies have been developed for OC, but the outcomes have not proved satisfactory. The new generation of immunotherapies has also been used to some extent for OC patients [14]. For instance, Avastin (bevacizumab), a recombinant antibody targeting VEGF/VEGFR signaling in tumor microenvironment, has been approved by the Food and Drug Administration (FDA) for the treatment of certain women with the advanced OC [15]. However, other immunotherapies in OC remain largely unknown, which mainly attributes to its highly heterogeneous nature [7]. Unlike other cancers, the expression of programmed cell death ligand 1 (PD-L1) in OC is not positively correlated to the efficacy of programmed cell death-1 (PD-1) inhibitors [16].

Insights into the importance of extracellular vesicles (EVs) in cancers have developed rapidly [17–19]. EVs are divided into different groups based on their sizes and mechanisms of biogenesis. The major groups that are widely investigated include exosomes (40–160 nm in diameter, endosomal origin), ectosomes (100–1000 nm in diameter, direct budding of the plasma membrane, also known as microparticles/microvesicles), apoptotic bodies (1–5 μm in diameter), large oncosomes (1–10 μm), and other miscellaneous EV subsets [20–22]. Among these groups, exosomes have drawn the most attention due to their unique biogenesis and function. Exosomes contain proteins, DNAs, lipids, and both coding and noncoding RNAs (ncRNAs), while ncRNAs consist of microRNAs (miRNAs), long noncoding RNAs (lncRNAs) and circular RNAs (circRNAs). According to the International Society of Extracellular Vesicles (ISEV), the term “extracellular vesicles” is the appropriate terminology for heterogeneous populations of vesicles isolated from cell culture supernatants or physiological fluids [23, 24]. Throughout this review, exosomes are referred to as EVs.

Accumulating evidence indicates that EVs play important roles in the progression, metastasis, and drug resistance of OC [25–27]. Fundamental research efforts have been put on investigating the application of EVs as a diagnostic biomarker as well as exploiting EVs as a drug-delivery vehicle in OC [28]. Moreover, the role of EVs as immunotherapy also represents a new developing area of research [29]. Here, we review the latest findings regarding the roles of EVs in OC chemoresistance, metastasis, and immune evasion. We also discuss the challenges of EV research that must be met if EVs are to be exploited to develop new diagnostic and therapeutic strategies for OC treatment.

## EVs and OC chemoresistance

Chemoresistance is the main challenge in the current OC treatment [30]. The proposed mechanisms of OC chemoresistance are multifaceted with contributory factors attributed to cancer stem cells (CSCs), ncRNAs, autophagy, DNA repair barriers, hypoxia and other changes in the tumor microenvironment (TME). Platinum or paclitaxel derivatives combined with surgical reduction are the standard first-line treatment strategy for OC [31]. Although most patients initially respond to platinum-based chemotherapy, about 70–80% of tumors recur and become resistant to treatment, especially the high-grade serous ovarian carcinoma (HGSOC) histological subtype [4]. Therefore, due to the high recurrence and chemotherapy-resistance rates, the 5-year survival rates of stage III and IV patients are 42 and 26%, respectively, according to the HGSOC staging of the International Federation of Obstetrics and Gynecology (FIGO) [32]. Thus, based on the perspective of conventional chemotherapy, finding novel therapies to overcome drug resistance is urgently needed.

As described above, EVs carry a mixed cargo of biological effectors and there is increasing evidence that these contribute to drug resistance in OC. Notably, the number of EVs secreted by cisplatin-resistant OC cells was 2.6 times that of drug-sensitive cells [33]. Moreover, the more aggressive OC cell line is, the more EVs they secrete [34]. Through gene ontology analysis, 12 proteins related to cell migration in SKOV3 cell derived EVs with stronger invasive ability were identified compared with the EV proteins in OVCAR3 cells, such as hepatocyte growth factor receptor (HGFR), integrin alpha-V (ITGAV) [34], suggesting that EVs from OC cells contain a specific set of proteins that are representative of its cell of origin and the invasive capacity. In addition, the analysis of plasma EVs of patients with OC also found that the number of EVs and protein content were more abundant than that in the healthy-control group [35, 36]. Drug-resistant cells can transmit resistance contents to sensitive cells through EVs [37]. Taken together, these findings propose an inherent link among EVs, tumor phenotype, drug treatment, and resistance.

**Fig. 1:**
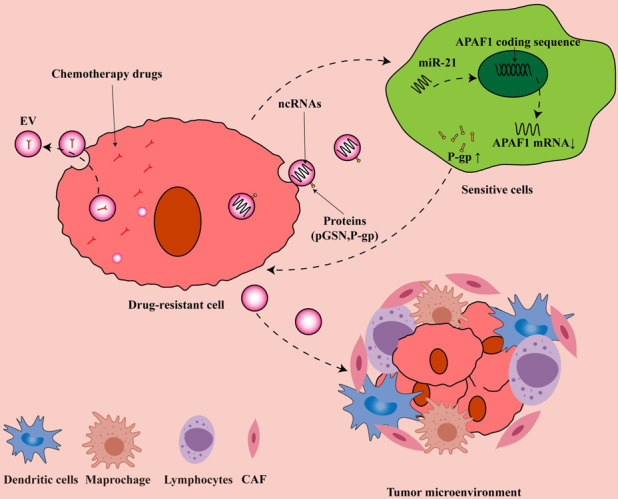
EVs-mediated drug-resistance mechanism of OC. Drug-resistant cells excrete chemotherapeutic drugs out of the cell by secreting EVs to achieve the purpose of chemotherapy resistance. At the same time, the ncRNA and active protein contained in the EVs secreted by the drug-resistant cells enter the sensitive cells through endocytosis, thereby transmitting the drug-resistant phenotype. We use EV miR-21 [49] as an example to demonstrate the emergence of this resistance mechanism. EVs secreted by drug-resistant cells also change the tumor microenvironment, making tumor cells more likely to survive.

One instructive study found that the EVs produced by treating OC cells with cisplatin promoted both the platinum resistance and the invasiveness of cells [38]. In another study, plasma gelsolin (pGSN) transported by EVs conferred OC cells resistance to cis-diamminedichloroplatinum (CDDP) via inducing CD8 T-cell apoptosis and promoting glutathione (GSH) production [39]. Regarding the former observation, it was found that P-glycoprotein (P-gp), which caused chemoresistance in patients with OC, was transported between cells by EVs and increased the resistance of A2780 cells to adriamycin and paclitaxel by five times in vitro [40]. High expression of GATA3 in EVs of ascites from HGSOC patients was reported to induce drug resistance in OVCAR3 cells [41]. The STAT3 and FAS oncoproteins carried by EVs secreted by OC cells isolated from the patients’ ascites significantly increased the resistance to cisplatin and the ability of cell migration in vitro [25]. Finally, increased levels of plasma gelsolin, a form of gelsolin secreted in EVs by OC cells, correlate with poor outcomes in patients and were shown to act in an autocrine and paracrine manner to confer platinum resistance to OC cells [4]. Along with these protein examples, miRNAs carried by EVs are also involved in the development of OC chemoresistance [42]. Here, we summarize the relationship between EVs and chemoresistance in OC in Fig. 1.

### EV miRNAs in OC chemoresistance

Due to the important roles of EV cargo in tumors, previous studies systematically explored EV cargo, including DNAs, mRNAs, lipids and proteins, about its functions in cancer detection [43–45]. The excellent performance of miRNAs in regulating changes of cell phenotype, cytokine expression, and secretion has become a research hotspot in TME [46]. In OC, miRNAs encapsulated in EVs can be protected from enzyme ribonuclease, which is related to platinum resistance and recurrence [47]. Therefore, EV miRNAs could be used as a biomarker to monitor OC chemoresistance [47, 48].

A number of in vitro studies have shown that when “harmful” EV miRNAs are taken up by tumor cells, chemoresistance will further develop. For example, Alharbi Mona et al. found that EV miR-21-5p activates glycolysis and increases the expression of the ATP-binding cassette family and detoxification enzymes, thereby promoting carboplatin resistance in OC cells [47]. In this study, the authors also showed that miR-891-5p helps OC cells gain platinum resistance by increasing the expression of tumor suppressor P53 (TP53) and X-ray repair cross-complementing 5 (XRCC5), resulting in altered control of the G2/M-checkpoint proteins, which can arrest the cell cycle when DNA is damaged, thereby providing time for cell DNA repair [47]. Another study found that cancer-associated fibroblasts (CAFs) and cancer-associated adipocyte (CAA)-derived EVs isolated from HGSOC patients carry miR-21, which lowers the expression of apoptotic protease-activating factor 1 (APAF1) in OVCA432 and SKOV3 cells, resulting in insensitivity to paclitaxel [49]. EVs secreted by CAFs also exert significant biological effects with transferring miR-98-5p to OC cells and promoting cisplatin resistance by targeting the expression of CDKN1A [50]. Nonetheless, there are also reports indicating that EVs carrying miR-30a-5p reduce the resistance of SKOV3 cells by targeting inhibition of SRY box-9 (SOX9) expression [51].

Some recent studies have found that EV miRNAs also play important roles in chemoresistance of OC via different signaling pathways. EV miRNAs, including miR-223 [52], miR-1246 [53], miR-433 [54], miR-21 [49], and miR-1307 [55], have been shown to promote OC cell resistance to paclitaxel by different pathways. For instance, EV miR-223 induces chemoresistance in SKOV3 cells through the PTEN–PI3K/AKT pathway. MiR-1246 inhibits the Cav1 gene and affects the recipient cells through the PDGFβ receptor, while miR-433 induces senescence of OC cells. EV miR-98-5p [50], miR-130a [56], miR-21-3p [37], miR-891-5p [47], miR-223 [52], miR-214-3p [57], and miR-214 [58] have all been shown to be associated with platinum resistance in OC cells. In contrast, miR-100 [59], miR-143 [60], and miR-30a-5p [51] in EVs can induce OC cells to resensitize to platinum. Table 1 illustrates the effects of some highly expressed EV miRNAs on chemoresistance in OC cell lines.

**Table 1:** Summary of EV miRNAs in OC chemoresistance.

miRNA	OC cell line	Drug	Possible mechanism	Reference
miR-21	OVCA432, SKOV3	Paclitaxel	miR-21 downregulates the expression of APAF1	[48]
miR-1246	HeyA8, SKOV3, A2780	Paclitaxel	Cav1/p-gp/M2-type macrophage axis	[52]
miR-433	A2780, PEO1, PEO4	Paclitaxel	Induce cellular senescence	[53]
miR-1307	A2780	Paclitaxel	miR-1307 downregulates the expression of ING5	[54]
miR-21-5p	CAOV3	Cisplatin	Glycolysis /ATP-binding cassette family and detoxification enzymes	[46]
miR-21-3p	CAOV3	Cisplatin	Upregulate the synthesis of glutathione synthase, NUCL, TPD53, MATR3 and XRCC5	[46]
miR-891-5p	CAOV3	Cisplatin	Increase the expression of G2/M checkpoints	[46]
miR-98-5p	SKOV3	Cisplatin	miR-98-5p /CDKN1A, inhibit apoptosis	[49]
miR-223	A2780, SKOV3	Cisplatin	miR-223 inhibits the expression of PTEN to activate the PI3K/AKT pathway	[51]
miR-214	A2780	Cisplatin	cell apoptosis	[57]
miR-214-3p	OV90, ES2	Cisplatin, Paclitaxel	miR-214-3p inhibits the expression of LHX6	[56]
miR-1270	A2780, SKOV3	Cisplatin	miR-1270 downregulates the expression of SCAI	[66]
miR-143	A2780, SKOV3	↓Cisplatin	miR-143 downregulates the expression of FOSL2	[59]
miR-30a-5p	SKOV3	↓Cisplatin	miR-30a-5p downregulates the expression of SOX9	[50]
miR-100	SKOV3	↓Cisplatin	Inhibit cell proliferation, promote cell apoptosis and cell cycle arrest	[58]

*Abbreviations***:** AKT also known as PKB, protein kinase B, APAF1 apoptotic protease-activating factor 1, CDKN1A cyclin dependent kinase inhibitor 1A, EV extracellular vesicles, FOSL2 Fos-like antigen 2, ING5 inhibitor of growth 5, LHX6 LIM homeobox 6, MATR3 matrin 3, NUCL nucleolar protein, PI3K phosphatidylinositol 3-kinase, PTEN Phosphatase and tensin homolog, SCAI the suppressor of cancer cell invasion, SOX9 SRY box 9, TPD53 tumor P53, XRCC5 X-ray repair cross-complementation 5. ↓ Reversed the resistance of OC cells to chemotherapy drugs.

Thus, from these studies, it is evident that EVs are not only triggered by chemotherapy drugs but their mixed complement of cargo molecules directly contributes to the chemoresistant phenotype of OC cells. Overall, we classify the effects of EVs and chemoresistance into four categories. First, OC cells directly excrete drug molecules by secreting EVs; second, EVs participate in the development of chemoresistance by transporting drug-resistance-related ncRNA; third, EVs improve the tolerance of target cells to chemotherapeutic drugs by transporting active proteins; finally, EVs alter the chemoresistance of OC cells by regulating the TME. The results of these studies provide the basis for exploring the future directions for EVs as biomarkers as well as the basis for novel anticancer treatments, as discussed further below.

Last, it must be mentioned that OC chemoresistance mechanisms involving EVs do not stand alone since there are many other contributing factors, involving changes in cell metabolism and epigenetics [61, 62].

### EV noncoding RNA biomarkers for monitoring OC chemoresistance

EVs found in plasma or serum fractions are not only derived from blood cells but also from all body tissues. Some articles have proposed the utility of EVs as circulating biomarkers for monitoring OC chemoresistance [4, 53, 63, 64]. All major types of EV cargo have been considered, but unsurprisingly, miRNAs feature prominently as potential analytes, given the abundance of studies involving EV miRNAs.

Several studies have found that the high expression of miRNAs in circulating EVs is associated with platinum resistance and recurrence of OC [47, 57]. For instance, miR-891a-5p was found to be highly expressed in patients with recurrent OC (*n* = 6) compared with patients that were alive without disease (*n* = 11). Epithelial ovarian cancer (EOC) cells transfected with miR-891a-5p increased the expression of G2/M-checkpoint proteins involved in the DNA repair mechanism, which might be related to the indication of platinum resistance and high risk of relapse [47]. Another study examined the expression of EV miRNAs from the serum of 29 patients after primary debulking surgery, miR-214-3p was found to be positively correlated with EOC malignancy. The target gene of EV miR-214-3p is LHX6. The inhibition of LHX6 reduced the rate of EOC cell apoptosis induced by cisplatin, suggesting that LHX6 is associated with resistance to platinum-based chemotherapy [57]. EV miR-223 and miR-484 are also proposed to predict the chemoresistance and prognosis of OC patients [52, 65]. The expression of EV miR-223 was significantly higher than that of patients with primary tumors [52], in a group of OC patients (*n* = 12) who received paclitaxel and cisplatin chemotherapy after the first surgery and relapsed within 6 months. In another study, the researchers examined the levels of EV miR-484 in the serum of OC patients (*n* = 113) and healthy individuals (*n* = 60), and found that the expression of serum EV miR-484 in OC patients was significantly reduced. At the same time, they found that the low expression of serum EV miR-484 usually predicts shorter OS and PFS [65]. However, the above studies are all based on findings in cells, which revealed potential mechanisms that can be used in determining patients’ responses.

Other forms of ncRNAs have also been considered as EV biomarkers to monitor the progression and chemoresistance of OC. The high expression of lncRNA MALAT1 and lncRNA UCA1 in serum EVs usually indicates that OC is at an advanced stage and the occurrence of cisplatin resistance [60, 66]. CircRNA Foxp1 was upregulated in serum EVs of cisplatin-resistant OC patients [67], in contrast, circRNA Cdr1as was downregulated [68]. In platinum-resistant OC cells, the significantly increased expression of EV pGSN has been proposed as a biomarker of chemoresistance [4]. The EV noncoding RNA biomarkers proposed for monitoring OC chemoresistance are summarized in Table 2.

**Table 2:** EV noncoding RNA biomarkers proposed for monitoring OC chemoresistance.

Cargo type	Sample origin	EV cargo	Pathway/Mode of action	Reference
MiRNA	Serum	MiR-214-3p	Inhibit the expression of LHX6	[56]
	Plasma	MiR-891-5p	Increase the expression of G2/M checkpoints	[46]
	Serum	MiR-484	Regulate angiogenesis	[63]
	Macrophages	MiR-223	Inhibit the expression of PTEN to activate the PI3K/AKT pathway	[51]
LncRNA	SKOV3.ip1, HO8910.PM	LncRNA MALAT1	Regulate angiogenesis	[64]
	Serum	LncRNA UCA1	Inhibit the expression of miR-143 thereby upregulating FOSL2	[59]
CircRNA	Serum	CircRNA Cdr1as	Inhibit the expression of miR-1270thereby upregulating SCAI	[66]
	SKOV3	CircRNA Foxp1	Upregulate the expression of CEBPG and FMNL3 through miR-22 and miR-150-3p	[65]

*Abbreviations:* AKT also known as PKB, protein kinase B; Cdr1as cerebellar degeneration-related protein 1 antisense RNA, CEBPG CCAAT enhancer binding protein gamma, EV extracellular vesicles, FMNL3 formin like 3, FOSL2 Fos-likeantigen2, Foxp1 forkhead box P1, LHX6 LIM homeobox 6, MALAT1 metastasis associated lung adenocarcinoma transcript 1, PI3K phosphatidylinositol 3-kinase, PTEN Phosphatase and tensin homolog, UCA1 urothelial cancer associated 1.

## EVs in OC metastasis

**Fig. 2:**
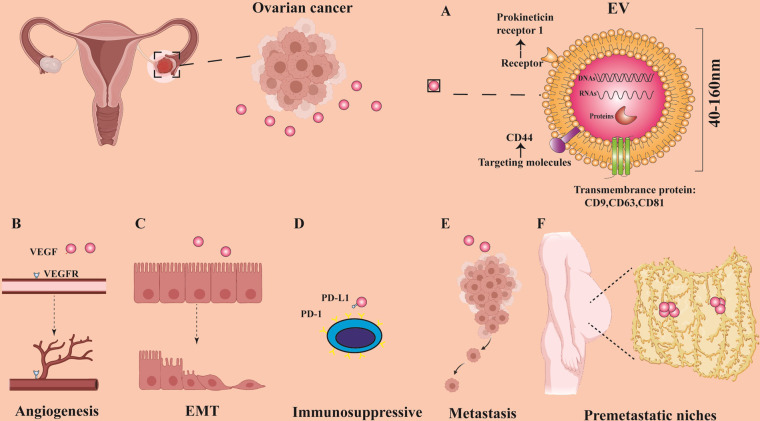
The role of EVs in OC metastasis. OC-derived EVs promote OC metastasis by promoting angiogenesis, affecting the EMT process, producing immunosuppression, directly stimulating OC cells, and participating in the construction of premetastatic niches. **A** It shows the general structure, size, content, and markers of EVs [22, 24]. **B** The VEGF carried by EVs binds to the VEGFR of vascular endothelial cells to promote angiogenesis [79]. **C** EVs promote the process of EMT [84]. **D** PD-L1 is expressed on the surface of EVs, which inhibits the activation of immune cells [120]. **E** EVs directly stimulate OC cells, causing them to metastasize [25]. **F** EVs are involved in the construction of the niche before OC peritoneal metastasis [96].

The role of EVs in cell communication has been widely recognized [22, 69]. Tumor metastasis is also closely related to the secretion of EVs [70, 71]. In Table 3, we summarize some EV cargos related to OC metastasis. Sharma Shayna et al. confirmed that 12 proteins related to proliferation, invasion, and metastasis exist in EVs secreted by SKOV3 cells [34]. Others found that EV-mediated metastasis of cancer-secreting miR-105 disrupts the integrity of the vascular endothelial barrier [72]. In contrast, miR-6126 [73] and miR-7 [74] in EVs were shown to inhibit the proliferation and metastasis of OC. Analogously, EVs in human adipose mesenchymal stem cell (hAMSC)-derived conditioned medium (CM) were shown to inhibit the proliferation of A2780 and SKOV3 OC cells [75]. In addition, SKOV3-derived EVs promoted the transformation of normal stromal fibroblasts into CAFs in vitro, and CAFs in turn enhanced the migration ability of SKOV3 cells [76]. EVs are thought to stimulate angiogenesis, extracellular matrix (ECM) remodeling, establish premetastatic niches, inhibit immune response, and promote tumor metastasis. The role of EVs in OC metastasis promoted by the different factors is shown in Fig. 2.

**Table 3:** EV cargos related to OC metastasis.

Function	EV cargo	Original cell	Recipient cell	Signaling pathways/Mechanism	Reference
Angiogenesis	MiR-141-3p	SKOV3	Endothelial cell	Activate the JAK-STAT3 signaling pathway and upregulate the expression of VEGFR-2	[79]
	MiR-205	HO-8910, SKOV3	Endothelial cell	Silence PTEN activates the AKT signaling pathway and upregulating the expression of VEGFA	[80, 81]
	LncRNA ENST00000444164, LncRNA ENST0000043768	SKOV3	HUVECs	NF-κB	[83]
	CD147	A2780, OVCAR3, SKOV3	HUVECs	CD147/MMP	[85]
	PKR1	A2780, HO8910	HUVECs	STAT3	[86]
	sE-cad	CAOV3, OV90	HUVECs	NF-κB	[87]
Targeted metastasis	CD44	HeyA8, TYK-nu	Peritoneal mesothelial cells	CD44/MMP9	[94]
	ACTN4, type IV collagen	SKOV3	N/A	Wnt/β-catenin	[95]
	L1CAM, CD24, ADAM10, EMMPRIN	SKOV3, malignant ascites (*n* = 3)	N/A	N/A	[25]
	MMP1 mRNA	ES-2, malignant ascites (*n* = 6)	Peritoneal mesothelial cells	Apoptosis	[43]
	CircRNA PUM1	A2780, CAOV3	Peritoneal mesothelial cells	NF-κB, MMP2	[97]
	CircRNA WHSC1	CAOV3, OVCAR3	Peritoneal mesothelial cells	Regulate MUC1 and hTERT	[98]

*Abbreviations*: ACTN4 actinin alpha 4, ADAM10 a disintegrin and metalloproteinase 10, CD cluster of differentiation, EMMPRIN extracellular matrix metalloproteinase inducer, hTERT human telomerase reverse transcriptase, HUVECs human umbilical vein endothelial cells, JAK janus kinase, L1CAM L1 cell adhesion molecule, MMP matrix metallopeptidase, MUC mucin, N/A not available, NF-κB nuclear factor kappa B, PKR1 prokineticin receptor 1, PUM1 pumilio RNA binding family member 1, sE-cad soluble E-cadherin, STAT3 signal transducer and activator of transcription 3, STAT3 signal transducer and activator of transcription 3, VEGFA vascular endothelial growth factor A, VEGFR2 vascular endothelial growth factor receptor 2, WHSC1 Wolf–Hirschhorn syndrome candidate 1.

### EVs affect the regulation of OC angiogenesis

As we know, blood vessels not only provide oxygen and nutrients for the development of tumors, but are also considered essential for supporting tumor metastasis [77]. Indeed, the cargo molecules in tumor-derived EVs are involved in angiogenesis in vitro, which play an important role in promoting tumor metastasis [78, 79]. In clinical work, the combination of anti-angiogenic drugs such as Apatinib and Nintedanib and chemotherapy drugs can significantly improve the prognosis of OC patients [80, 81]. These anti-angiogenic drugs can selectively inhibit vascular endothelial growth-factor (VEGF) receptors. However, it is also reported that in certain cases, some can promote endothelial cells to release EVs enriched with VEGF to promote tumor angiogenesis [82]. EV MiR-141-3p secreted by OC cells was reported to promote the generation of vascular endothelial cells by activating the JAK/STAT3 signaling pathway, thereby accelerating the metastasis of OC [78]. Liuqing et al. found that circulating EV miR-205 was highly expressed in OC patients [79], and induced angiogenesis through the PTEN–AKT pathway, which had been shown to promote OC metastasis and invasion [79, 83]. In SKOV3 cells, miR-205 overexpressed in EVs can target vascular endothelial growth factor A (VEGF-A) to change its biological characteristics [84].

The lncRNA carried by EVs from SKOV3 cells can activate the phosphorylation of NF-κB in human umbilical vein endothelial cells (HUVECs), thereby promoting endothelial cell migration [85]. CD147 was found to be expressed in EVs derived from OVCAR3 OC cells, and CD147-positive vesicles released by OC cells could induce angiogenesis in vitro [86, 87]. Moreover, EV prokineticin-receptor 1 (PKR1) was shown to promote angiogenesis in A2780 and HO8910 OC cells in vitro and the mechanism may be related to the phosphorylation of STAT3 [88]. Soluble E-cadherin (sE-cad), which is located on the surface of EVs, is highly expressed in the malignant ascites of patients with OC and has been proven to be an effective angiogenesis inducer [89]. Most of these studies are limited to in vitro experiments, but demonstrate in principle that OC EVs regulate the formation of blood vessels in tumors.

### How do EVs promote OC metastasis?

Different tumor types tend to metastasize to specific sites, for example, prostate cancer mainly metastasizes to bone [90], while uveal melanoma usually metastasizes to the liver [91]. Cancer cells directly influence this process, for example, midkine secreted by cutaneous melanoma cells produces long-range changes in the endothelium, creating a premetastatic niche for lymphatic metastasis [92]. It has also been reported that tumor-derived EVs taken up by organ-specific cells similarly create a premetastatic niche [71]. Different EV cargoes, including proteins, nucleic acids, and lipids, are transported to specific organs, transforming the recipient’s tissue microenvironment, to assist the process of targeted metastasis [70, 93]. For OC, metastasis usually involves the spread of cancer cells to the omentum [49].

One of the fundamental ways in which EVs promote metastasis involves their ability to mediate cell–cell interactions [94]. For example, the cell-adhesion molecule CD44 that regulates the process of OC metastasis in an organ-specific manner [95], was reported to be transferred to the peritoneal mesothelial cells from OC cells via EVs to assist in their invasion [96]. Alharbi Mona et al. found that the EVs secreted by SKOV3 cells were rich in proteins that regulated cancer signaling through ACTN4, CD44, and type-IV collagen [97]. ACTN4 is an actin-binding protein that plays a key role in the movement of cancer cells [98]. There is also evidence that sE-cad-positive EVs are associated with the formation of malignant ascites and the extensive peritoneal metastasis of OC cells [89]. Keller Sascha et al. found that the EVs derived from malignant ascites carry proteins related to tumor progression, such as L1CAM, CD24, ADAM10, and EMMPRIN [26]. Their animal experiments proved that using EVs derived from malignant ascites to stimulate tumor-bearing mice caused the tumor to spread in the abdomen [26]. RNA-type EV cargos also influence targeted metastasis in OC. For instance, MMP1 mRNA carried by EVs leads to OC peritoneal metastasis by inducing apoptosis of human mesothelial cells [44]. The EV circRNA CircPUM1 and circWHSC1 promote OC peritoneal metastasis through the sponging of miRNAs, which are essential for the mesothelial-to-mesenchymal transition (MMT) and epithelial-to-mesenchymal transition (EMT) processes of peritoneal mesothelial cells, respectively [99, 100].

Collectively, these studies suggest that EVs affect the metastasis of OC to the peritoneum. By acting on the peritoneum, EVs transform part of the microenvironment to make it suitable for the planting and growth of OC cells.

### Roles of EVs EMT and CSCs working together in promoting OC metastasis

The EMT is associated with cancer metastasis and resistance to treatment by enhancing cancer-cell plasticity [101]. In studies of OC, downregulation of TRAP1 evident in OC metastases is functionally associated with EMT and promoting OC invasion [102]. Furthermore, MUC4 mucin overexpression in ovarian tumors similarly induces EMT as in OC cells and enhances their invasiveness [103]. EVs also influence EMT through paracrine mechanisms [69], for example, miR-205 from OC-derived EVs regulates EMT in OC cells [84], while LIN28, an RNA-binding protein that is highly expressed in OC cell EVs, causes the increased EMT gene expression in HEK293 cells [104]. Ascite-derived EVs promote EMT by delivering miR-6780b-5p to OC cells [105]. The above results indicate that EVs regulate the process of OC EMT through multiple pathways.

Cancer stem cells (CSCs) are thought to be the core reason behind tumor clonal evolution [106], and thus widely believed to be the driving force behind tumor progression [107]. Cells with CSC characteristics have been isolated from OC cell lines [108, 109]. EMT is also thought to relate to tumor “stemness” and may represent a functional characteristic of CSCs [110]. In OC, CSCs can survive the first-line chemotherapy and develop drug-resistant metastases [111]. These CSCs are present in the ascites in the form of single or multicellular spheroids [112], and then disseminate to new locations with the ascites’ movement [111], forming extensive abdominal metastases. A study has shown that the EVs from MDA-MB-231 breast cancer cell line released miR-454 to disrupt the Wnt pathway, thereby promoting CSC stemness in SKOV3 and CoC1 OC cells in vitro [113]. This may imply that EVs and CSCs have certain relationship that jointly regulates the spread and resistance of OC. More deep investigation is required for the following study in this area.

## EVs influence OC immune evasion

**Fig. 3:**
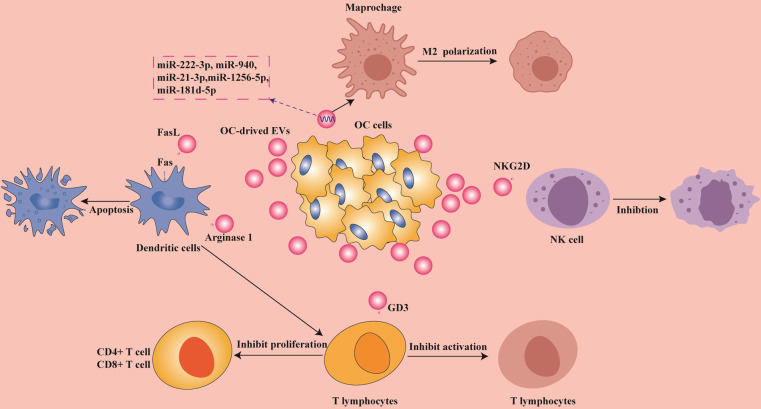
The role of EVs in OC immune evasion. OC-derived EVs inhibit the activation of DC cells, induce the polarization of macrophages, inhibit the cytotoxicity of NK cells, and regulate the function of T cells. The miRNAs carried in EVs secreted by OC cells promote the conversion of macrophages into M2 phenotype [124], and the FasL carried on the surface induces the apoptosis of DCs [128]. EV cargo also inhibits the proliferation of CD4^+^ and CD8^+^ T cells through the presentation of DCs [145]. EV cargo directly stimulates T cells and NK cells as well as inhibits their functional activation [136, 144]. EVs help OC cells produce immune evasion through these mechanisms of action.

With the advent of immune-checkpoint inhibitors, immunotherapy is reemerging as the next wave of promising anticancer treatments. The proportion of patients benefiting from immunotherapy in the clinic is still not high since tumors utilize multiple ways to escape the immune system [114]. The TME plays a very important role in cancer immune evasion, and comprises various immune and stromal cells that have profound effects on immune responses. Notably, cancer cells can escape immune surveillance by overexpression of antiphagocytic surface proteins such as CD47, PD-L1, and the beta-2 microglobulin subunits of major histocompatibility class-I (MHC-class-I) complex (B2M) [115]. OC usually has limited immune-cell infiltration or extensive immunosuppressive T-cell infiltration, making it a low immunoreactive cancer [116]. EVs are found to contribute to the process of immune evasion in a multifaceted manner that involves the effects on both the innate and adaptive immune response by regulating the function of immune cells [117]. For instance, PD-L1 expressed in EVs inhibits the antitumor immune response [118]. At the same time, the interaction between EV PD-L1 and PD-1 inhibits the function of T cells and promotes the adaptive immune escape of tumor [119, 120]. Immune evasion is usually associated with tumor metastasis. In Fig. 3, we show the potential role of EVs in regulating immune cells in OC. The following sections discuss how EVs contribute to immune escape in OC in detail.

### EVs contribute to macrophage phenotypic differentiation in OC

Tumor-associated macrophages (TAMs) are the most abundant immune cells in the TME [121]. They can be broadly categorized into two functional classes according to their polarization: inflammatory M1 and anti-inflammatory macrophage M2 phenotypes [121]. EOC cell-derived EV miR-940 plays a tumor-promoting role in EOC by driving macrophages to activate the M2 phenotype [122]. Macrophages of M2 phenotype activated by EVs help immune evasion of OC cells by releasing immunosuppressive factors [123]. EOC-derived EVs, including miR-21-3p, miR-125b-5p, and miR-181d-5p, have all been shown to induce M2 macrophage polarization, thereby promoting EOC occurrence and transfer [124]. TAM-derived EVs are rich in miR-29a-3p and miR-21-5p, which interact with T cells to create an immunosuppressive environment [125]. In addition, EVs isolated from ascites may also have a role in impairing the cytotoxic activity of peripheral blood mononuclear cells (PBMCs) [86]. Thus, there is abundant evidence from the above reports to suggest that EVs induce immune escape of OC by affecting the polarization of macrophages.

### Functions of EVs are associated with dendritic cells in OC

Dendritic cells (DCs) play a specialized role in antigen presentation that links the regulation of the innate and adaptive immune response. Several mechanisms have now been disclosed to show that cargo molecules in EVs produced by OC cells impact the function of DCs. For example, EV miR-212-3p promotes immune tolerance of DCs by inhibiting the expression of regulatory factor X-related protein (RFXAP) and major MHC class II [122]. Another study showed that OC EVs rich in miR-203 inhibit DC activation [126]. Tumor-derived EVs induced immunosuppression by delivering heat-shock proteins (HSP72 and HSP105) to DCs [127]. In vitro studies also showed that FAS-L and TRAIL expressed by OC-derived EVs in patients’ ascites inhibited DC activation by inducing apoptosis [128].

EVs produced by DCs have also been shown to express natural killer (NK) cell activation ligands and induce antigen-specific T- and B-cell responses [129]. Other recent research has confirmed that EVs are necessary for DCs to activate T-cell function [130]. Thus, OC-derived EVs inhibit the activation of DCs or EVs from the DCs act to suppress the immune response.

### Effects of tumor-derived EVs on NK cells

NK cells are a key antitumor effector cell that can spontaneously detect and lyse transformed or stressed cells [131]. Advanced pleural malignant mesothelioma cell-derived EVs were reported to inhibit the stimulation of IL-2 on lymphocyte proliferation, and significantly inhibited the cytotoxicity of NK cells [132]. Melanoma cell-derived EVs also downregulate NK-cell function and induce apoptosis in cytotoxic CD8^+^ T cells [133]. In contrast, in neuroblastoma, EVs secreted by NK cells restore the cytotoxic effects of NK cells [134].

Specifically, EVs from ascite-derived patients promote the progression of OC in vivo, and play a role in regulating immune responses in the TME. These EVs could also be taken up by NK and B cells [135]. OC-derived EVs expressing the NK receptor NKG2D ligands (MICA / B and ULBP1–3) were shown to interfere with NK-mediated tumor cell targeting [136], thereby reducing NK-cell function. Although there is presently limited evidence available, the fact that EVs antagonize NK function would seem to represent an obvious opportunity for improving the effectiveness of OC immunotherapy.

### EVs regulate lymphocyte function in tumor microenvironment

Regulatory T (Treg) cells play an important role in maintaining immune homeostasis, and are recruited in a large number in the TME, creating obstacles to tumor immunity [137]. OC-derived EVs suppress T-cell immunity and promote tumor progression [138, 139]. In a low-glucose environment, miR-451 encapsulated in gastric cancer-derived EVs was reported to promote the differentiation of T-helper 17 (Th17) cells [140]. It was found that melanoma-derived EVs reduced T-cell responses by reducing T-cell receptor (TCR) signaling and reducing cytokine and granzyme secreted by B cells, thereby reducing the cytotoxic activity of the cells [141]. Similarly, when hepatocellular carcinoma-derived EVs activated B cells, B cells showed the strong expression of the TIM-1 protein and inhibited CD8 T-cell activity [142]. In addition, EVs secreted by metastatic melanoma with PD-L1 expression inhibit CD8 T-cell function and promote tumor growth [120]. The abundant contents in EVs also mediate the transfer of OC cells through interaction with immune cells.

A range of specific inhibitory mechanisms from OC-derived EVs have recently been reported. T cells treated with EVs derived from OC ascites are functionally suppressed and interestingly, the inhibitory effect is reversible if exposed to EVs for a short time (2 h), but longer exposures (1–4 days) result in the gradually but irreversible loss of T-cell function [143]. GD3, a ganglioside expressed on the surface of OC EVs, directly inhibited the activation of T cells [144]. The effects were also observed in the lymphatics where EVs from OC are dispersed long distance to draining lymph nodes and transfer arginase 1 to DCs, thereby inhibiting the proliferation of CD4^+^ and CD8^+^ T cells [145]. Furthermore, OC-derived EVs help promote immune escape by inhibiting the release of IL-2 and IFN-γ of CD4^+^ and CD8^+^ T cells [143]. Moreover, siglec-10 on the surface of T cells is upregulated by EVs secreted from OC cells, and acts to inhibit T-cell activation [146]. All the results indicate that OC cells regulate the function of T lymphocytes through the contents of EVs to achieve the purpose of immune evasion.

### The potential application of EVs in OC immunotherapy

As described above, EVs produced by tumor cells have a variety of negative effects on the immune response. Equally, there are some reports showing that the EVs isolated from ascites of OC patients stimulate cytokine secretion by monocytes that may enhance immune responses [147]. Conceptually therefore, using EVs to reactivate anti-tumor immunity, e.g., pancreatic cancer-cell EVs reverting macrophages M2 to an M1 phenotype, may represent an alternative mode of treatment [148]. Conejo-Garcia and his team verified that FSHR (follicle stimulating hormone receptors) are a safe and effective immunotherapy target for the treatment of OC, and proposed that the FSHR carried in ascite-derived EVs specifically activate FSHCER (follicle-stimulating hormone receptor chimeric endocrine receptor) T cells [149]. Another opportunity may involve manipulating the potential of EVs to alter antigen-presenting functions in the TME [150]. EVs released from OC ascites present antigens to DCs and may activate cytotoxic T cells [151]. Therefore, the antigen-presentation capability of EVs may provide a new direction for the development of OC vaccines. However, further research is necessary to study the related mechanisms in more detail, especially to identify specific EVs and/or EV cargo that contribute to enhancing immunotherapy or other treatment options for OC.

## Novel therapeutic approaches using EVs for OC treatment

Chemoresistance is currently the main difficulty in clinical treatment of OC and EVs play various roles in the manifestation of drug resistance. Researchers in the United States have proposed a device, similar to a hemodialysis machine, that can separate circulating EVs from the body to reduce the circulating levels of EVs, with the idea to suppress the development of drug resistance in tumor cells [152]. This bold concept is yet to be proven, but in support, GW4869, which is a pharmacological agent that inhibits EV generation, was shown to reverse the resistance of drug-resistant OC cells and restored its sensitivity to cisplatin [153].

In addition, to be a potential therapeutic target, EVs can also be used as carriers for drugs and other forms of therapies [28]. Jin et al. reported that EVs secreted by breast cancer or OC contributed to the pharmacodynamics between nearby cells where they transfered drugs from one cell to another [154]. In this application, EVs have the advantages of low immunogenicity, better biocompatibility, and targeted efficacy [155]. For example, Mohamad et al. found that encapsulating doxorubicin with EVs in mice avoided cardiotoxicity and improved the therapeutic effect on OC [156]. Moreover, Xiaohui et al. transferred cisplatin into EVs, which significantly improved the cytotoxic effect of cisplatin in drug-resistant A2780/DDP cells in vitro [157]. EVs can also be adapted to mimic their natural effects on gene regulation. EVs secreted by OC designed by Chenglong and colleagues carrying both MDR1–siRNA and paclitaxel were shown to restore sensitivity to paclitaxel both in vitro and in vivo [158]. Most recently, Simone et al. described immune derived exosome mimetics (IDEM) that carried chemotherapeutic drugs. Compared with natural EVs, this system has higher yields, lower side effects, and more precise targeting [159]. Similarly, Jang et al. designed an EV loaded with the STimulator of InterferoN Genes (STING) agonist cyclic dinucleotide (CDN), named ExoSTING, which had a 100–200-fold increase in effectiveness and could effectively activate immune cells [160]. Therefore, while using EVs to treat chemoresistance in OC has not yet reached the clinic, progress has been made to identify both the right targets and the approaches to deliver the EVs.

## Conclusions and perspectives

The resistance, metastasis, and immune evasion of OC are highly correlated with EVs, which provides a new idea for clinical management of this disease. In this review, we describe the role of EVs and their contents in OC resistance, abdominal metastasis, and immune evasion. In view of various problems in the clinical management of OC, many scientific research teams have conducted a number of investigations related to EVs, which provides new insights and theoretical guidance for future clinical treatment. In the meantime, there are also some unsolved problems in the study of EVs. For example, the gold standard for EV isolation has not been established, and clinical testing is not practically used. The obstacles for EVs from the laboratory research to clinical transformation also exist. With the deepening understanding on EVs, it may be available to indicate the prognosis of patients based on the expression of EV contents, and develop the personalized treatment strategies to benefit OC patients in the future.

## Data Availability

The datasets analyzed during the current review are available from the corresponding authors on reasonable request.

## References

[1] Sung H, Ferlay J, Siegel RL, Laversanne M, Soerjomataram I, Jemal A (2021). Global cancer statistics 2020: GLOBOCAN estimates of incidence and mortality worldwide for 36 cancers in 185 countries. CA Cancer J Clin.

[2] Siegel RL, Miller KD, Jemal A (2020). Cancer statistics, 2020. CA Cancer J Clin.

[3] Cao W, Chen HD, Yu YW, Li N, Chen WQ (2021). Changing profiles of cancer burden worldwide and in China: a secondary analysis of the global cancer statistics 2020. Chin Med J.

[4] Asare-Werehene M, Nakka K, Reunov A, Chiu CT, Lee WT, Abedini MR (2020). The exosome-mediated autocrine and paracrine actions of plasma gelsolin in ovarian cancer chemoresistance. Oncogene.

[5] Jayson GC, Kohn EC, Kitchener HC, Ledermann JA (2014). Ovarian cancer. Lancet.

[6] Muinao T, Pal M, Deka (2018). Boruah HP. Origins based clinical and molecular complexities of epithelial ovarian cancer. Int J Biol Macromol.

[7] Lheureux S, Braunstein M, Oza AM (2019). Epithelial ovarian cancer: Evolution of management in the era of precision medicine. CA Cancer J Clin.

[8] Cecere SC, Giannone G, Salutari V, Arenare L, Lorusso D, Ronzino G (2020). Olaparib as maintenance therapy in patients with BRCA 1-2 mutated recurrent platinum sensitive ovarian cancer: Real world data and post progression outcome. Gynecol Oncol.

[9] Pujade-Lauraine E, Ledermann JA, Selle F, Gebski V, Penson RT, Oza AM (2017). Olaparib tablets as maintenance therapy in patients with platinum-sensitive, relapsed ovarian cancer and a BRCA1/2 mutation (SOLO2/ENGOT-Ov21): a double-blind, randomised, placebo-controlled, phase 3 trial. Lancet Oncol.

[10] Poveda A, Floquet A, Ledermann JA, Asher R, Penson RT, Oza AM (2021). Olaparib tablets as maintenance therapy in patients with platinum-sensitive relapsed ovarian cancer and a BRCA1/2 mutation (SOLO2/ENGOT-Ov21): a final analysis of a double-blind, randomised, placebo-controlled, phase 3 trial. Lancet Oncol.

[11] Aabo K, Adams M, Adnitt P, Alberts DS, Athanazziou A, Barley V (1998). Chemotherapy in advanced ovarian cancer: four systematic meta-analyses of individual patient data from 37 randomized trials. Advanced Ovarian Cancer Trialists’ Group. Br J Cancer.

[12] Eisenkop SM, Friedman RL, Spirtos NM (2000). The role of secondary cytoreductive surgery in the treatment of patients with recurrent epithelial ovarian carcinoma. Cancer.

[13] Clarke-Pearson DL, DeLong ER, Chin N, Rice R, Creasman WT (1988). Intestinal obstruction in patients with ovarian cancer. Variables associated with surgical complications and survival. Arch Surg.

[14] Lee JM, Minasian L, Kohn EC (2019). New strategies in ovarian cancer treatment. Cancer.

[15] Arora S, Balasubramaniam S, Zhang H, Berman T, Narayan P, Suzman D (2021). FDA approval summary: Olaparib monotherapy or in combination with bevacizumab for the maintenance treatment of patients with advanced ovarian cancer. Oncologist.

[16] Coleman RL (2016). Ovarian cancer in 2015: Insights into strategies for optimizing ovarian cancer care. Nat Rev Clin Oncol.

[17] Minciacchi VR, Freeman MR, Di (2015). Vizio D. Extracellular vesicles in cancer: exosomes, microvesicles and the emerging role of large oncosomes. Semin Cell Dev Biol.

[18] Mathew M, Zade M, Mezghani N, Patel R, Wang Y, Momen-Heravi F (2020). Extracellular vesicles as biomarkers in cancer immunotherapy. Cancers.

[19] Walker S, Busatto S, Pham A, Tian M, Suh A, Carson K (2019). Extracellular vesicle-based drug delivery systems for cancer treatment. Theranostics.

[20] Margolis L, Sadovsky Y (2019). The biology of extracellular vesicles: The known unknowns. PLoS Biol.

[21] Cocucci E, Meldolesi J (2015). Ectosomes and exosomes: shedding the confusion between extracellular vesicles. Trends Cell Biol.

[22] Kalluri R, LeBleu VS (2020). The biology, function, and biomedical applications of exosomes. Science.

[23] Witwer KW, Buzas EI, Bemis LT, Bora A, Lasser C, Lotvall J, et al. Standardization of sample collection, isolation and analysis methods in extracellular vesicle research. *J Extracell Vesicles* 2: 10.3402/jev.v2i0.20360 (2013). 10.3402/jev.v2i0.20360 PMC3760646 24009894

[24] Thery C, Witwer KW, Aikawa E, Alcaraz MJ, Anderson JD, Andriantsitohaina R (2018). Minimal information for studies of extracellular vesicles 2018 (MISEV2018): a position statement of the International Society for Extracellular Vesicles and update of the MISEV2014 guidelines. J Extracell Vesicles.

[25] Dorayappan KDP, Wanner R, Wallbillich JJ, Saini U, Zingarelli R, Suarez AA (2018). Hypoxia-induced exosomes contribute to a more aggressive and chemoresistant ovarian cancer phenotype: a novel mechanism linking STAT3/Rab proteins. Oncogene.

[26] Keller S, Konig AK, Marme F, Runz S, Wolterink S, Koensgen D (2009). Systemic presence and tumor-growth promoting effect of ovarian carcinoma released exosomes. Cancer Lett.

[27] Kobayashi M, Salomon C, Tapia J, Illanes SE, Mitchell MD, Rice GE (2014). Ovarian cancer cell invasiveness is associated with discordant exosomal sequestration of Let-7 miRNA and miR-200. J Transl Med.

[28] Tran PHL, Xiang D, Tran TTD, Yin W, Zhang Y, Kong L (2020). Exosomes and nanoengineering: A match made for precision therapeutics. Adv Mater.

[29] Jella KK, Nasti TH, Li Z, Malla SR, Buchwald ZS, Khan MK (2018). Exosomes, their biogenesis and role in inter-cellular communication, tumor microenvironment and cancer immunotherapy. Vaccines.

[30] Li ZY, Wang XL, Dang Y, Zhu XZ, Zhang YH, Cai BX (2020). Long non-coding RNA UCA1 promotes the progression of paclitaxel resistance in ovarian cancer by regulating the miR-654-5p/SIK2 axis. Eur Rev Med Pharm Sci.

[31] Armstrong DK, Bundy B, Wenzel L, Huang HQ, Baergen R, Lele S (2006). Intraperitoneal cisplatin and paclitaxel in ovarian cancer. N. Engl J Med.

[32] Torre LA, Trabert B, DeSantis CE, Miller KD, Samimi G, Runowicz CD (2018). Ovarian cancer statistics, 2018. CA Cancer J Clin.

[33] Safaei R, Larson BJ, Cheng TC, Gibson MA, Otani S, Naerdemann W (2005). Abnormal lysosomal trafficking and enhanced exosomal export of cisplatin in drug-resistant human ovarian carcinoma cells. Mol Cancer Ther.

[34] Sharma S, Alharbi M, Kobayashi M, Lai A, Guanzon D, Zuniga F (2018). Proteomic analysis of exosomes reveals an association between cell invasiveness and exosomal bioactivity on endothelial and mesenchymal cell migration in vitro. Clin Sci.

[35] Zhang W, Ou X, Wu X (2019). Proteomics profiling of plasma exosomes in epithelial ovarian cancer: A potential role in the coagulation cascade, diagnosis and prognosis. Int J Oncol.

[36] Kabe Y, Suematsu M, Sakamoto S, Hirai M, Koike I, Hishiki T (2018). Development of a highly sensitive device for counting the number of disease-specific exosomes in human sera. Clin Chem.

[37] Pink RC, Samuel P, Massa D, Caley DP, Brooks SA, Carter DR (2015). The passenger strand, miR-21-3p, plays a role in mediating cisplatin resistance in ovarian cancer cells. Gynecol Oncol.

[38] Samuel P, Mulcahy LA, Furlong F, McCarthy HO, Brooks SA, Fabbri M (2018). Cisplatin induces the release of extracellular vesicles from ovarian cancer cells that can induce invasiveness and drug resistance in bystander cells. Philos Trans R Soc Lond B Biol Sci.

[39] Asare-Werehene M, Communal L, Carmona E, Han Y, Song YS, Burger D (2020). Plasma Gelsolin inhibits CD8(+) T-cell function and regulates glutathione production to confer chemoresistance in ovarian cancer. Cancer Res.

[40] Zhang FF, Zhu YF, Zhao QN, Yang DT, Dong YP, Jiang L (2014). Microvesicles mediate transfer of P-glycoprotein to paclitaxel-sensitive A2780 human ovarian cancer cells, conferring paclitaxel-resistance. Eur J Pharm.

[41] El-Arabey AA, Denizli M, Kanlikilicer P, Bayraktar R, Ivan C, Rashed M (2020). GATA3 as a master regulator for interactions of tumor-associated macrophages with high-grade serous ovarian carcinoma. Cell Signal.

[42] Santos P, Almeida F (2020). Role of exosomal miRNAs and the tumor microenvironment in drug resistance. Cells.

[43] Keseru JS, Soltesz B, Lukacs J, Marton E, Szilagyi-Bonizs M, Penyige A (2019). Detection of cell-free, exosomal and whole blood mitochondrial DNA copy number in plasma or whole blood of patients with serous epithelial ovarian cancer. J Biotechnol.

[44] Yokoi A, Yoshioka Y, Yamamoto Y, Ishikawa M, Ikeda SI, Kato T (2017). Malignant extracellular vesicles carrying MMP1 mRNA facilitate peritoneal dissemination in ovarian cancer. Nat Commun.

[45] Cheng L, Zhang K, Qing Y, Li D, Cui M, Jin P (2020). Proteomic and lipidomic analysis of exosomes derived from ovarian cancer cells and ovarian surface epithelial cells. J Ovarian Res.

[46] Rupaimoole R, Calin GA, Lopez-Berestein G, Sood AK (2016). miRNA Deregulation in Cancer Cells and the Tumor Microenvironment. Cancer Disco.

[47] Alharbi M, Sharma S, Guanzon D, Lai A, Zuniga F, Shiddiky MJA (2020). miRNa signature in small extracellular vesicles and their association with platinum resistance and cancer recurrence in ovarian cancer. Nanomedicine.

[48] Taylor DD, Gercel-Taylor C (2008). MicroRNA signatures of tumor-derived exosomes as diagnostic biomarkers of ovarian cancer. Gynecol Oncol.

[49] Au Yeung CL, Co NN, Tsuruga T, Yeung TL, Kwan SY, Leung CS (2016). Exosomal transfer of stroma-derived miR21 confers paclitaxel resistance in ovarian cancer cells through targeting APAF1. Nat Commun.

[50] Guo H, Ha C, Dong H, Yang Z, Ma Y, Ding Y (2019). Cancer-associated fibroblast-derived exosomal microRNA-98-5p promotes cisplatin resistance in ovarian cancer by targeting CDKN1A. Cancer Cell Int.

[51] Liu R, Zhang Y, Sun P, Wang C (2020). DDP-resistant ovarian cancer cells-derived exosomal microRNA-30a-5p reduces the resistance of ovarian cancer cells to DDP. Open Biol.

[52] Zhu X, Shen H, Yin X, Yang M, Wei H, Chen Q (2019). Macrophages derived exosomes deliver miR-223 to epithelial ovarian cancer cells to elicit a chemoresistant phenotype. J Exp Clin Cancer Res.

[53] Kanlikilicer P, Bayraktar R, Denizli M, Rashed MH, Ivan C, Aslan B (2018). Exosomal miRNA confers chemo resistance via targeting Cav1/p-gp/M2-type macrophage axis in ovarian cancer. EBioMedicine.

[54] Weiner-Gorzel K, Dempsey E, Milewska M, McGoldrick A, Toh V, Walsh A (2015). Overexpression of the microRNA miR-433 promotes resistance to paclitaxel through the induction of cellular senescence in ovarian cancer cells. Cancer Med.

[55] Chen WT, Yang YJ, Zhang ZD, An Q, Li N, Liu W (2017). MiR-1307 promotes ovarian cancer cell chemoresistance by targeting the ING5 expression. J Ovarian Res.

[56] Li Z, Yan-Qing W, Xiao Y, Shi-Yi L, Meng-Qin Y, Shu X (2021). Exosomes secreted by chemoresistant ovarian cancer cells promote angiogenesis. J Ovarian Res.

[57] Yang C, Kim HS, Park SJ, Lee EJ, Kim SI, Song G (2019). Inhibition of miR-214-3p aids in preventing epithelial ovarian cancer malignancy by increasing the expression of LHX6. Cancers.

[58] Zhang J, Liu J, Xu X, Li L (2017). Curcumin suppresses cisplatin resistance development partly via modulating extracellular vesicle-mediated transfer of MEG3 and miR-214 in ovarian cancer. Cancer Chemother Pharm.

[59] Pan C, Stevic I, Muller V, Ni Q, Oliveira-Ferrer L, Pantel K (2018). Exosomal microRNAs as tumor markers in epithelial ovarian cancer. Mol Oncol.

[60] Li Z, Niu H, Qin Q, Yang S, Wang Q, Yu C (2019). lncRNA UCA1 mediates resistance to cisplatin by regulating the miR-143/FOSL2-signaling pathway in ovarian cancer. Mol Ther Nucleic Acids.

[61] Gentric G, Kieffer Y, Mieulet V, Goundiam O, Bonneau C, Nemati F (2019). PML-regulated mitochondrial metabolism enhances chemosensitivity in human ovarian cancers. Cell Metab.

[62] Lin KC, Lin MW, Hsu MN, Yu-Chen G, Chao YC, Tuan HY (2018). Graphene oxide sensitizes cancer cells to chemotherapeutics by inducing early autophagy events, promoting nuclear trafficking and necrosis. Theranostics.

[63] Vasconcelos MH, Caires HR, Abols A, Xavier CPR, Line A (2019). Extracellular vesicles as a novel source of biomarkers in liquid biopsies for monitoring cancer progression and drug resistance. Drug Resist Updat.

[64] Li T, Lin L, Liu Q, Gao W, Chen L, Sha C (2021). Exosomal transfer of miR-429 confers chemoresistance in epithelial ovarian cancer. Am J Cancer Res.

[65] Zhang W, Su X, Li S, Liu Z, Wang Q, Zeng H (2020). Low serum exosomal miR-484 expression predicts unfavorable prognosis in ovarian cancer. Cancer Biomark.

[66] Qiu JJ, Lin XJ, Tang XY, Zheng TT, Lin YY, Hua KQ (2018). Exosomal metastasisassociated lung adenocarcinoma transcript 1 promotes angiogenesis and predicts poor prognosis in epithelial ovarian cancer. Int J Biol Sci.

[67] Luo Y, Gui R (2020). Circulating exosomal circFoxp1 confers cisplatin resistance in epithelial ovarian cancer cells. J Gynecol Oncol.

[68] Zhao Z, Ji M, Wang Q, He N, Li Y (2019). Circular RNA Cdr1as upregulates SCAI to suppress cisplatin resistance in ovarian cancer via miR-1270 suppression. Mol Ther Nucleic Acids.

[69] Becker A, Thakur BK, Weiss JM, Kim HS, Peinado H, Lyden D (2016). Extracellular vesicles in cancer: cell-to-cell mediators of metastasis. Cancer Cell.

[70] Wortzel I, Dror S, Kenific CM, Lyden D (2019). Exosome-mediated metastasis: communication from a distance. Dev Cell.

[71] Hoshino A, Costa-Silva B, Shen TL, Rodrigues G, Hashimoto A, Tesic Mark M (2015). Tumour exosome integrins determine organotropic metastasis. Nature.

[72] Zhou W, Fong MY, Min Y, Somlo G, Liu L, Palomares MR (2014). Cancer-secreted miR-105 destroys vascular endothelial barriers to promote metastasis. Cancer Cell.

[73] Kanlikilicer P, Rashed MH, Bayraktar R, Mitra R, Ivan C, Aslan B (2016). Ubiquitous release of exosomal tumor suppressor miR-6126 from ovarian cancer cells. Cancer Res.

[74] Hu Y, Li D, Wu A, Qiu X, Di W, Huang L (2017). TWEAK-stimulated macrophages inhibit metastasis of epithelial ovarian cancer via exosomal shuttling of microRNA. Cancer Lett.

[75] Reza A, Choi YJ, Yasuda H, Kim JH (2016). Human adipose mesenchymal stem cell-derived exosomal-miRNAs are critical factors for inducing anti-proliferation signalling to A2780 and SKOV-3 ovarian cancer cells. Sci Rep.

[76] Giusti I, Di Francesco M, D’Ascenzo S, Palmerini MG, Macchiarelli G, Carta G (2018). Ovarian cancer-derived extracellular vesicles affect normal human fibroblast behavior. Cancer Biol Ther.

[77] Cassetta L, Pollard JW (2018). Targeting macrophages: therapeutic approaches in cancer. Nat Rev Drug Disco.

[78] Masoumi-Dehghi S, Babashah S, Sadeghizadeh M (2020). microRNA-141-3p-containing small extracellular vesicles derived from epithelial ovarian cancer cells promote endothelial cell angiogenesis through activating the JAK/STAT3 and NF-kappaB signaling pathways. J Cell Commun Signal.

[79] He L, Zhu W, Chen Q, Yuan Y, Wang Y, Wang J (2019). Ovarian cancer cell-secreted exosomal miR-205 promotes metastasis by inducing angiogenesis. Theranostics.

[80] Lan CY, Wang Y, Xiong Y, Li JD, Shen JX, Li YF (2018). Apatinib combined with oral etoposide in patients with platinum-resistant or platinum-refractory ovarian cancer (AEROC): a phase 2, single-arm, prospective study. Lancet Oncol.

[81] du Bois A, Kristensen G, Ray-Coquard I, Reuss A, Pignata S, Colombo N (2016). Standard first-line chemotherapy with or without nintedanib for advanced ovarian cancer (AGO-OVAR 12): a randomised, double-blind, placebo-controlled phase 3 trial. Lancet Oncol.

[82] Zeng Y, Yao X, Liu X, He X, Li L, Liu X (2019). Anti-angiogenesis triggers exosomes release from endothelial cells to promote tumor vasculogenesis. J Extracell Vesicles.

[83] Li J, Hu K, Gong G, Zhu D, Wang Y, Liu H (2017). Upregulation of MiR-205 transcriptionally suppresses SMAD4 and PTEN and contributes to human ovarian cancer progression. Sci Rep.

[84] Wang L, Zhao F, Xiao Z, Yao L (2019). Exosomal microRNA-205 is involved in proliferation, migration, invasion, and apoptosis of ovarian cancer cells via regulating VEGFA. Cancer Cell Int.

[85] Wu Q, Wu X, Ying X, Zhu Q, Wang X, Jiang L (2017). Suppression of endothelial cell migration by tumor associated macrophage-derived exosomes is reversed by epithelial ovarian cancer exosomal lncRNA. Cancer Cell Int.

[86] Li X, Wang X (2017). The emerging roles and therapeutic potential of exosomes in epithelial ovarian cancer. Mol Cancer.

[87] Millimaggi D, Mari M, D’Ascenzo S, Carosa E, Jannini EA, Zucker S (2007). Tumor vesicle-associated CD147 modulates the angiogenic capability of endothelial cells. Neoplasia.

[88] Zhang X, Sheng Y, Li B, Wang Q, Liu X, Han J (2021). Ovarian cancer derived PKR1 positive exosomes promote angiogenesis by promoting migration and tube formation in vitro. Cell Biochem Funct.

[89] Tang MKS, Yue PYK, Ip PP, Huang RL, Lai HC, Cheung ANY (2018). Soluble E-cadherin promotes tumor angiogenesis and localizes to exosome surface. Nat Commun.

[90] Dai J, Escara-Wilke J, Keller JM, Jung Y, Taichman RS, Pienta KJ (2019). Primary prostate cancer educates bone stroma through exosomal pyruvate kinase M2 to promote bone metastasis. J Exp Med.

[91] Salarian M, Yang H, Turaga RC, Tan S, Qiao J, Xue S (2019). Precision detection of liver metastasis by collagen-targeted protein MRI contrast agent. Biomaterials.

[92] Olmeda D, Cerezo-Wallis D, Riveiro-Falkenbach E, Pennacchi PC, Contreras-Alcalde M, Ibarz N (2017). Whole-body imaging of lymphovascular niches identifies pre-metastatic roles of midkine. Nature.

[93] Liu Y, Cao X (2016). Organotropic metastasis: role of tumor exosomes. Cell Res.

[94] Steinbichler TB, Dudas J, Riechelmann H, Skvortsova II (2017). The role of exosomes in cancer metastasis. Semin Cancer Biol.

[95] Sacks Suarez J, Gurler Main H, Muralidhar GG, Elfituri O, Xu HL, Kajdacsy-Balla AA (2019). CD44 regulates formation of spheroids and controls organ-specific metastatic colonization in epithelial ovarian carcinoma. Mol Cancer Res.

[96] Nakamura K, Sawada K, Kinose Y, Yoshimura A, Toda A, Nakatsuka E (2017). Exosomes promote ovarian cancer cell invasion through transfer of CD44 to peritoneal mesothelial cells. Mol Cancer Res.

[97] Alharbi M, Lai A, Guanzon D, Palma C, Zuniga F, Perrin L (2019). Ovarian cancer-derived exosomes promote tumour metastasis in vivo: an effect modulated by the invasiveness capacity of their originating cells. Clin Sci.

[98] Shao H, Wang JH, Pollak MR, Wells A (2010). alpha-actinin-4 is essential for maintaining the spreading, motility and contractility of fibroblasts. PLoS ONE.

[99] Guan X, Zong ZH, Liu Y, Chen S, Wang LL, Zhao Y (2019). circPUM1 promotes tumorigenesis and progression of ovarian cancer by sponging miR-615-5p and miR-6753-5p. Mol Ther Nucleic Acids.

[100] Zong ZH, Du YP, Guan X, Chen S, Zhao Y (2019). CircWHSC1 promotes ovarian cancer progression by regulating MUC1 and hTERT through sponging miR-145 and miR-1182. J Exp Clin Cancer Res.

[101] Ishay-Ronen D, Diepenbruck M, Kalathur RKR, Sugiyama N, Tiede S, Ivanek R (2019). Gain fat-lose metastasis: converting invasive breast cancer cells into adipocytes inhib cancer metastasis. Cancer Cell.

[102] Amoroso MR, Matassa DS, Agliarulo I, Avolio R, Lu H, Sisinni L (2016). TRAP1 downregulation in human ovarian cancer enhances invasion and epithelial-mesenchymal transition. Cell Death Dis.

[103] Ponnusamy MP, Lakshmanan I, Jain M, Das S, Chakraborty S, Dey P (2010). MUC4 mucin-induced epithelial to mesenchymal transition: a novel mechanism for metastasis of human ovarian cancer cells. Oncogene.

[104] Enriquez VA, Cleys ER, Da Silveira JC, Spillman MA, Winger QA, Bouma GJ (2015). High LIN28A expressing ovarian cancer cells secrete exosomes that induce invasion and migration in HEK293 cells. Biomed Res Int.

[105] Cai J, Gong L, Li G, Guo J, Yi X, Wang Z (2021). Exosomes in ovarian cancer ascites promote epithelial-mesenchymal transition of ovarian cancer cells by delivery of miR-6780b-5p. Cell Death Dis.

[106] Vermeulen L, de Sousa e Melo F, Richel DJ, Medema JP (2012). The developing cancer stem-cell model: clinical challenges and opportunities. Lancet Oncol.

[107] Putzer BM, Solanki M, Herchenroder O (2017). Advances in cancer stem cell targeting: How to strike the evil at its root. Adv Drug Deliv Rev.

[108] Szotek PP, Pieretti-Vanmarcke R, Masiakos PT, Dinulescu DM, Connolly D, Foster R (2006). Ovarian cancer side population defines cells with stem cell-like characteristics and Mullerian Inhibiting Substance responsiveness. Proc Natl Acad Sci USA.

[109] Li J, Condello S, Thomes-Pepin J, Ma X, Xia Y, Hurley TD (2017). Lipid desaturation is a metabolic marker and therapeutic target of ovarian cancer stem cells. Cell Stem Cell.

[110] Marcucci F, Ghezzi P, Rumio C (2017). The role of autophagy in the cross-talk between epithelial-mesenchymal transitioned tumor cells and cancer stem-like cells. Mol Cancer.

[111] Zong X, Nephew KP (2019). Ovarian cancer stem cells: Role in metastasis and opportunity for therapeutic targeting. Cancers.

[112] Lengyel E (2010). Ovarian cancer development and metastasis. Am J Pathol.

[113] Wang L, He M, Fu L, Jin Y (2020). Exosomal release of microRNA-454 by breast cancer cells sustains biological properties of cancer stem cells via the PRRT2/Wnt axis in ovarian cancer. Life Sci.

[114] Goldberg MS (2019). Improving cancer immunotherapy through nanotechnology. Nat Rev Cancer.

[115] Barkal AA, Brewer RE, Markovic M, Kowarsky M, Barkal SA, Zaro BW (2019). CD24 signalling through macrophage Siglec-10 is a target for cancer immunotherapy. Nature.

[116] Majidpoor J, Mortezaee K (2021). The efficacy of PD-1/PD-L1 blockade in cold cancers and future perspectives. Clin Immunol.

[117] Ingenito F, Roscigno G, Affinito A, Nuzzo S, Scognamiglio I, Quintavalle C (2019). The role of Exo-miRNAs in cancer: A focus on therapeutic and diagnostic applications. Int J Mol Sci.

[118] Daassi D, Mahoney KM, Freeman GJ (2020). The importance of exosomal PDL1 in tumour immune evasion. Nat Rev Immunol.

[119] You L, Wu W, Wang X, Fang L, Adam V, Nepovimova E (2020). The role of hypoxia-inducible factor 1 in tumor immune evasion. Med Res Rev.

[120] Chen G, Huang AC, Zhang W, Zhang G, Wu M, Xu W (2018). Exosomal PD-L1 contributes to immunosuppression and is associated with anti-PD-1 response. Nature.

[121] Chanmee T, Ontong P, Konno K, Itano N (2014). Tumor-associated macrophages as major players in the tumor microenvironment. Cancers.

[122] Wang M, Yu F, Ding H, Wang Y, Li P, Wang K (2019). Emerging function and clinical values of exosomal microRNAs in cancer. Mol Ther Nucleic Acids.

[123] Cheng H, Wang Z, Fu L, Xu T (2019). Macrophage polarization in the development and progression of ovarian cancers: An overview. Front Oncol.

[124] Chen X, Zhou J, Li X, Wang X, Lin Y, Wang X (2018). Exosomes derived from hypoxic epithelial ovarian cancer cells deliver microRNAs to macrophages and elicit a tumor-promoted phenotype. Cancer Lett.

[125] Zhou J, Li X, Wu X, Zhang T, Zhu Q, Wang X (2018). Exosomes released from tumor-associated macrophages transfer miRNAs that induce a Treg/Th17 cell imbalance in epithelial ovarian cancer. Cancer Immunol Res.

[126] Klibi J, Niki T, Riedel A, Pioche-Durieu C, Souquere S, Rubinstein E (2009). Blood diffusion and Th1-suppressive effects of galectin-9-containing exosomes released by Epstein-Barr virus-infected nasopharyngeal carcinoma cells. Blood.

[127] Shen Y, Guo D, Weng L, Wang S, Ma Z, Yang Y (2017). Tumor-derived exosomes educate dendritic cells to promote tumor metastasis via HSP72/HSP105-TLR2/TLR4 pathway. Oncoimmunology.

[128] Peng P, Yan Y, Keng S (2011). Exosomes in the ascites of ovarian cancer patients: origin and effects on anti-tumor immunity. Oncol Rep.

[129] Gehrmann U, Naslund TI, Hiltbrunner S, Larssen P, Gabrielsson S (2014). Harnessing the exosome-induced immune response for cancer immunotherapy. Semin Cancer Biol.

[130] Ruhland MK, Roberts EW, Cai E, Mujal AM, Marchuk K, Beppler C (2020). Visualizing synaptic transfer of tumor antigens among dendritic cells. Cancer Cell.

[131] Huntington ND, Cursons J, Rautela J (2020). The cancer-natural killer cell immunity cycle. Nat Rev Cancer.

[132] Clayton A, Mitchell JP, Court J, Mason MD, Tabi Z (2007). Human tumor-derived exosomes selectively impair lymphocyte responses to interleukin-2. Cancer Res.

[133] Sharma P, Diergaarde B, Ferrone S, Kirkwood JM, Whiteside TL (2020). Melanoma cell-derived exosomes in plasma of melanoma patients suppress functions of immune effector cells. Sci Rep.

[134] Schmittgen TD (2019). Exosomal miRNA cargo as mediator of immune escape mechanisms in neuroblastoma. Cancer Res.

[135] Rodriguez GM, Galpin KJC, McCloskey CW, Vanderhyden BC (2018). The tumor microenvironment of epithelial ovarian cancer and its influence on response to immunotherapy. Cancers.

[136] Cai X, Caballero-Benitez A, Gewe MM, Jenkins IC, Drescher CW, Strong RK (2017). Control of tumor initiation by NKG2D naturally expressed on ovarian cancer cells. Neoplasia.

[137] Watson MJ, Vignali PDA, Mullett SJ, Overacre-Delgoffe AE, Peralta RM, Grebinoski S (2021). Metabolic support of tumour-infiltrating regulatory T cells by lactic acid. Nature.

[138] Lobb RJ, Lima LG, Moller A (2017). Exosomes: Key mediators of metastasis and pre-metastatic niche formation. Semin Cell Dev Biol.

[139] Taylor DD, Gercel-Taylor C, Lyons KS, Stanson J, Whiteside TL (2003). T-cell apoptosis and suppression of T-cell receptor/CD3-zeta by Fas ligand-containing membrane vesicles shed from ovarian tumors. Clin Cancer Res.

[140] Huang T, Song C, Zheng L, Xia L, Li Y, Zhou Y (2019). The roles of extracellular vesicles in gastric cancer development, microenvironment, anti-cancer drug resistance, and therapy. Mol Cancer.

[141] Vignard V, Labbe M, Marec N, Andre-Gregoire G, Jouand N, Fonteneau JF (2020). MicroRNAs in tumor exosomes drive immune escape in melanoma. Cancer Immunol Res.

[142] Ye L, Zhang Q, Cheng Y, Chen X, Wang G, Shi M (2018). Tumor-derived exosomal HMGB1 fosters hepatocellular carcinoma immune evasion by promoting TIM-1(+) regulatory B cell expansion. J Immunother Cancer.

[143] Shenoy GN, Loyall J, Maguire O, Iyer V, Kelleher RJ, Minderman H (2018). Exosomes associated with human ovarian tumors harbor a reversible checkpoint of T-cell responses. Cancer Immunol Res.

[144] Shenoy GN, Loyall J, Berenson CS, Kelleher RJ, Iyer V, Balu-Iyer SV (2018). Sialic acid-dependent inhibition of T cells by exosomal ganglioside GD3 in ovarian tumor microenvironments. J Immunol.

[145] Czystowska-Kuzmicz M, Sosnowska A, Nowis D, Ramji K, Szajnik M, Chlebowska-Tuz J (2019). Small extracellular vesicles containing arginase-1 suppress T-cell responses and promote tumor growth in ovarian carcinoma. Nat Commun.

[146] Li Y, Zhou J, Zhuo Q, Zhang J, Xie J, Han S (2019). Malignant ascite-derived extracellular vesicles inhibit T cell activity by upregulating Siglec-10 expression. Cancer Manag Res.

[147] Rashed MH, Kanlikilicer P, Rodriguez-Aguayo C, Pichler M, Bayraktar R, Bayraktar E (2017). Exosomal miR-940 maintains SRC-mediated oncogenic activity in cancer cells: a possible role for exosomal disposal of tumor suppressor miRNAs. Oncotarget.

[148] Su MJ, Aldawsari H, Amiji M (2016). Pancreatic cancer cell exosome-mediated macrophage reprogramming and the role of microRNAs 155 and 125b2 transfection using nanoparticle delivery systems. Sci Rep.

[149] Perales-Puchalt A, Svoronos N, Rutkowski MR, Allegrezza MJ, Tesone AJ, Payne KK (2017). Follicle-stimulating hormone receptor is expressed by most ovarian cancer subtypes and is a safe and effective immunotherapeutic target. Clin Cancer Res.

[150] Tang MK, Wong AS (2015). Exosomes: Emerging biomarkers and targets for ovarian cancer. Cancer Lett.

[151] Li QL, Bu N, Yu YC, Hua W, Xin XY (2008). Exvivo experiments of human ovarian cancer ascites-derived exosomes presented by dendritic cells derived from umbilical cord blood for immunotherapy treatment. Clin Med Oncol.

[152] Marleau AM, Chen CS, Joyce JA, Tullis RH (2012). Exosome removal as a therapeutic adjuvant in cancer. J Transl Med.

[153] Cao YL, Zhuang T, Xing BH, Li N, Li Q (2017). Exosomal DNMT1 mediates cisplatin resistance in ovarian cancer. Cell Biochem Funct.

[154] Wang J, Yeung BZ, Cui M, Peer CJ, Lu Z, Figg WD (2017). Exosome is a mechanism of intercellular drug transfer: Application of quantitative pharmacology. J Control Release.

[155] Liu C, Zhang W, Li Y, Chang J, Tian F, Zhao F (2019). Microfluidic sonication to assemble exosome membrane-coated nanoparticles for immune evasion-mediated targeting. Nano Lett.

[156] Hadla M, Palazzolo S, Corona G, Caligiuri I, Canzonieri V, Toffoli G (2016). Exosomes increase the therapeutic index of doxorubicin in breast and ovarian cancer mouse models. Nanomed.

[157] Zhang X, Liu L, Tang M, Li H, Guo X, Yang X (2020). The effects of umbilical cord-derived macrophage exosomes loaded with cisplatin on the growth and drug resistance of ovarian cancer cells. Drug Dev Ind Pharm.

[158] Wang C, Guan W, Peng J, Chen Y, Xu G, Dou H (2020). Gene/paclitaxel co-delivering nanocarriers prepared by framework-induced self-assembly for the inhibition of highly drug-resistant tumors. Acta Biomater.

[159] Pisano S, Pierini I, Gu J, Gazze A, Francis LW, Gonzalez D (2020). Immune (cell) derived exosome mimetics (IDEM) as a treatment for ovarian cancer. Front Cell Dev Biol.

[160] Jang SC, Economides KD, Moniz RJ, Sia CL, Lewis N, McCoy C (2021). ExoSTING, an extracellular vesicle loaded with STING agonists, promotes tumor immune surveillance. Commun Biol.

